# Synthesis of Silica-coated Iron Oxide Nanoparticles: Preventing Aggregation without Using Additives or Seed Pretreatment

**Published:** 2018

**Authors:** Zeinab Sharafi, Bita Bakhshi, Jaber Javidi, Sina Adrangi

**Affiliations:** a *Department of Pharmaceutical Biotechnology, School of Pharmacy, Shahid Beheshti University of Medical Sciences, Tehran, Iran.*; b *Department of Medical Bacteriology, Faculty of Medical Sciences, Tarbiat Modares University, Tehran, Iran. *; c *Department of Pharmaceutics and Nanotechnology, School of Pharmacy, Shahid Beheshti University of Medical Sciences, Tehran, Iran.*

**Keywords:** Nanoparticle, Magnetite, Silica, Sol-gel, Aggregation

## Abstract

The Stober process is frequently used to prepare silica-coated iron oxide nanoparticles. This is usually achieved by seeding a reaction mixture consisting of water, ethanol and a catalyst with iron oxide particles and adding a silica precursor. The hydrolysis and condensation of precursor monomers results in the deposition of a silica layer on iron oxide particles. However, this process is accompanied by an increase in the ionic strength of the medium which promotes the rapid aggregation of iron oxide particles. A number of methods have been developed to prevent seed aggregation during the coating process. The majority of these methods include a pretreatment step in which the surface of iron oxide particles is modified in a manner that increases their stability in aqueous solutions. Here we suggest that by decreasing the initial concentration of the catalyst for a short period to minimize nucleation by reducing precursor hydrolysis rate and then gradually increasing the concentration to the optimum level to allow silica formation to proceed normally it may be possible to prevent aggregation without surface modification. The properties of the resulting nanoparticles as analyzed by transmission electron microscopy and magnetometry as well as their efficiency at extracting genomic DNA from different bacterial strains compared to that of a commercial extraction kit are also reported.

## Introduction

Nanoparticles (NPs) comprising a magnetic iron oxide core encapsulated in an appropriate organic or inorganic coat have been successfully used in a wide range of biomedical applications from macromolecule separation to magnetic resonance imaging (1-3). Among the various coating materials, silica is probably the best studied and most commonly used substance (1). The silica coat not only improves the stability of magnetic NPs in aqueous solutions, but also provides a scaffold for the attachment of different functional groups (4-6). Such silica-coated iron oxide (Fe_3_O_4_@SiO_2_) NPs can be prepared by several methods (4). One of the most widely employed methods for the synthesis of Fe_3_O_4_@SiO_2_ NPs is a procedure based on the sol-gel process described by Stober *et al*. (7). This method, known as the Stober process, was originally designed to prepare monodisperse suspensions of silica NPs through the alkaline hydrolysis and subsequent condensation of tetraethyl orthosilicate (TEOS) in a mixture of ethanol and water. It was later modified to produce Fe_3_O_4_@SiO_2_ NPs by seeding the reaction mixture with Fe_3_O_4_ nuclei (8). However, when added to the reaction mixture, Fe_3_O_4_ particles tend to aggregate and form large clusters entrapped in a silica matrix (6, 8). A number of approaches have been used to address this problem. These methods usually rely on the use of either an appropriate additive or some sort of pretreatment to improve the stability of Fe_3_O_4_ seeds in the water-ethanol reaction mixture. For example, Philipse *et al*. applied a thin layer of silica to Fe_3_O_4_ particles by exposing them to a dilute alkaline solution of sodium silicate for two hours before using them as seeds (8). Hui *et al*. used a combination of citrate and nitrate salts to produce hydrophilic citrate-capped Fe_3_O_4_ particles (9). Xu *et al*. also included a pretreatment step although in this case their primary goal was to increase the affinity of Fe_3_O_4_ particles to silica (5). Other methods such sonochemical and template-driven coating have also been reported in the literature (6, 10). Alternatively, some researchers have suggested that Fe_3_O_4_@SiO_2_ NPs can be obtained by coating the untreated Fe_3_O_4_ NPs under normal conditions and then separating aggregates with prolonged centrifugation at relatively high speeds (11). All these methods, despite their apparent advantages, introduce additional steps and thus increase the complexity of the process.

Although the mechanisms governing the formation of NPs in the Stober reaction are not completely understood (12, 13), it is known that the nature, concentration and molar ratios of reactants greatly influence the morphology and size of resulting particles (4, 7). For example, Bhakta *et al*. showed that decreasing the volumetric ratio of water to ethanol below certain levels results in the formation of a gel network rather than particulate structures (14). Similar results have also been reported for core-shell structures prepared by modifications of the Stober method. Using different chemical compounds as core-forming ligands, for example, Masse *et al*. showed that above certain ligand to TEOS ratios large particles with an increased tendency toward aggregation are formed while particles produced at lower ratios are noticeably more stable (12, 15). It may thus be hypothesized that by adjusting the reaction conditions during the coating process aggregate formation can be minimized or probably totally avoided. In this paper, we propose that by careful adjustments in the concentrations of reagents and the order and rate at which they are added to the reaction mixture it is possible to prevent aggregation without employing any additional procedures. Also, in order to evaluate the practicability of this approach, we compare the yield and quality of genomic DNA samples extracted from several bacterial strains using NPs produced by this method with those purified by a commercial DNA extraction kit.

## Experimental

*Reagents and bacterial strains*

All chemicals were used without further purification. FeCl_2_*.*4H_2_O, FeCl_3_*.*6H_2_O, TEOS, poly(ethylene glycol) (PEG, MW = 8000), NaCl, Tris-HCl, ethylene diaminetetraacetic acid (EDTA), sodium dodecyl sulphate (SDS), NaOH, HCl, calf thymus DNA (CT-DNA) were purchased from Sigma Aldrich (USA). EZ-10 Spin Column Bacterial DNA Mini-Preps Kit and proteinase K were purchased from Bio Basic (Canada). Primers for 16s rDNA amplification were synthesized by Boineer (Republic of Korea). RedSafe nucleic acid staining solution was purchased from iNtRON Biotechnology (Republic of Korea). *Bacillus licheniformis* was a kind gift from Dr. Faramarzi (School of Pharmacy, Tehran University of Medical Sciences). *Escherichia coli*, *Staphylococcus epidermidis*, *S. aureus*, *Yersinia enterocolitica*, and *Pseudomonas aeruginosa* were obtained from the culture collection of the Department of Medical Bacteriology of Tarbiat Modares University.

*Preparation of Fe*_3_ *O*_4_ *@SiO*_2_ *NPs*

The Fe_3_O_4_ NPs were prepared according to a protocol described earlier (16). Briefly, a mixture of FeCl_3_.6H_2_O (1.3 g), FeCl_2_*.*4H_2_O (0.48 g) and polyvinyl alcohol 15000 (300 mg) in 30 mL deionized water was heated at 80 °C for 30 min under N_2_ with vigorous stirring. After this period, 5 M NaOH (4.5 mL) was slowly added to produce a black precipitate. The mixture was then stirred at 60 °C for 2 h. The precipitate was collected using a permanent magnet and washed several times with deionized water and then absolute ethanol. The resulting Fe_3_O_4_ NPs were kept in ethanol. In order to apply the silica coat, Fe_3_O_4_ NPs (100 mg) were suspended in a mixture of anhydrous ethanol (80 mL) and deionized water (20 mL) and sonicated in a water bath for 5 min. Subsequently, 0.10 mL of TEOS was added under N_2_ and vigorous stirring and the dispersion was left under stirring at room temperature. After 10 min, 1.0 mL of NaOH solution (2 M) was added in 0.1 mL portions over a period of 2 h and the mixture was stirred for an additional 6 h at room temperature. Finally obtained Fe_3_O_4_@SiO_2_ NPs were separated by an external magnet, washed several times with deionized water and then absolute ethanol until the supernatant was clear and stored in ethanol until required.

*Characterization of Fe*_3_ *O*_4_ *@SiO*_2_ *NPs*

The size and morphology of magnetic NPs were investigated by transmission electron microscopy (TEM) using an EM10C*-*80 KV electron microscope (Zeiss, Germany)*.* The magnetic properties of NPs were evaluated by a BHV-55 vibrating sample magnetometer (VSM) (Riken Denshi, Japan) at room temperature.

*Determination of DNA binding and elution conditions using CT-DNA*

In order to determine binding conditions, CT-DNA (12μg), Fe_3_O_4_@SiO_2_ NPs (0.5 mg), NaCl (final concentration 0-2 M) and PEG 8000 (final concentration 0-20 % w/v) were added to 10 mM Tris-HCl buffer, pH 8 (final volume 100 μL) and incubated on a benchtop shaker at room temperature and 500 rpm for 5-45 min. Fe_3_O_4_@SiO_2_ NPs were then collected with an external magnet, washed twice with 50 μL of ethanol (90 %) and allowed to dry at 40 °C. For elution experiments, Fe_3_O_4_@SiO_2_ NPs from binding experiments (0.5 mg) were redispersed in 100 μL of TE buffer (10 mM Tris–HCl, 1 mM EDTA), pH 8.0 and incubated for 5 to 70 min at 50 °C. DNA concentrations were determined spectrophotometrically at 260 nm using a Synergy™ HTX multi-mode reader (BioTek Instruments Inc., USA).

The effect of pH on DNA adsorption was investigated by adding 0.5 mg Fe_3_O_4_@SiO_2_ NPs into a solution containing 12 μg CT-DNA in a 100 μL volume of an appropriate buffer (10 mM citrate buffer, pH 4-6; 10 mM Tris-HCl buffer, pH 7-9) and incubating the mixture at room temperature and 500 rpm for 30 min. The absorbance at 260 nm was used to estimate DNA concentration.

*Genomic DNA extraction*

Bacterial strains were inoculated into either nutrient broth, brain heart infusion broth or trypticase soy broth and incubated at 37 °C for 24 h. For each extraction experiment, 1.5 ml of the culture was transferred into a microtube and centrifuged at 10000*g* for 5 min. The pellet was resuspended in 500 μL of lysis buffer (10 mM TE buffer pH 8.0, 440 μL; 10 % SDS, 50 μL; proteinase K, 20 mg/mL, 10 μL) and incubated at 55 °C for 30 min. Subsequently, Fe_3_O_4_@SiO_2_ NPs (1 mg), NaCl (final concentration 1 M) and PEG 8000 (final concentration 5 % w/v) were added. The suspension was incubated at room temperature and 500 rpm for 25 min. The NPs were collected using a permanent magnet, washed twice with 200 μL of ethanol (90 %) and allowed to dry at 40 °C. DNA was eluted with 100 μL TE buffer (10 mM Tris–HCl, 1 mM EDTA), pH 8.0 at 50 °C for 60 min. Genomic DNA was also extracted using a commercial DNA extraction kit following the manufacturer’s standard protocol.

*Amplification of extracted DNA by PCR*

DNA samples extracted from bacterial strains by both methods were used as template for PCR amplification. A region of approximately 1500 pb of the 16S rDNA gene was amplified using primers 27F(5´-AGAGTTTGATCCTGGCTCAG-3´) and1(5´-TACGGCTACCTTGTTACGACTT-3´)(17). The PCR reactions were carried out in a peqSTARthermocycler(PEQLABBiotechnologie, Germany) using the following program: initial denaturation at 95 °C for 300 s, 30 cycles of denaturation at 95 °C for 60 s, annealing at 60 °C for 60 s and extension at 72 °C for 90 s and final extension at 72 °C for 600 s.

## Results and Discussion

**Figure 1:**
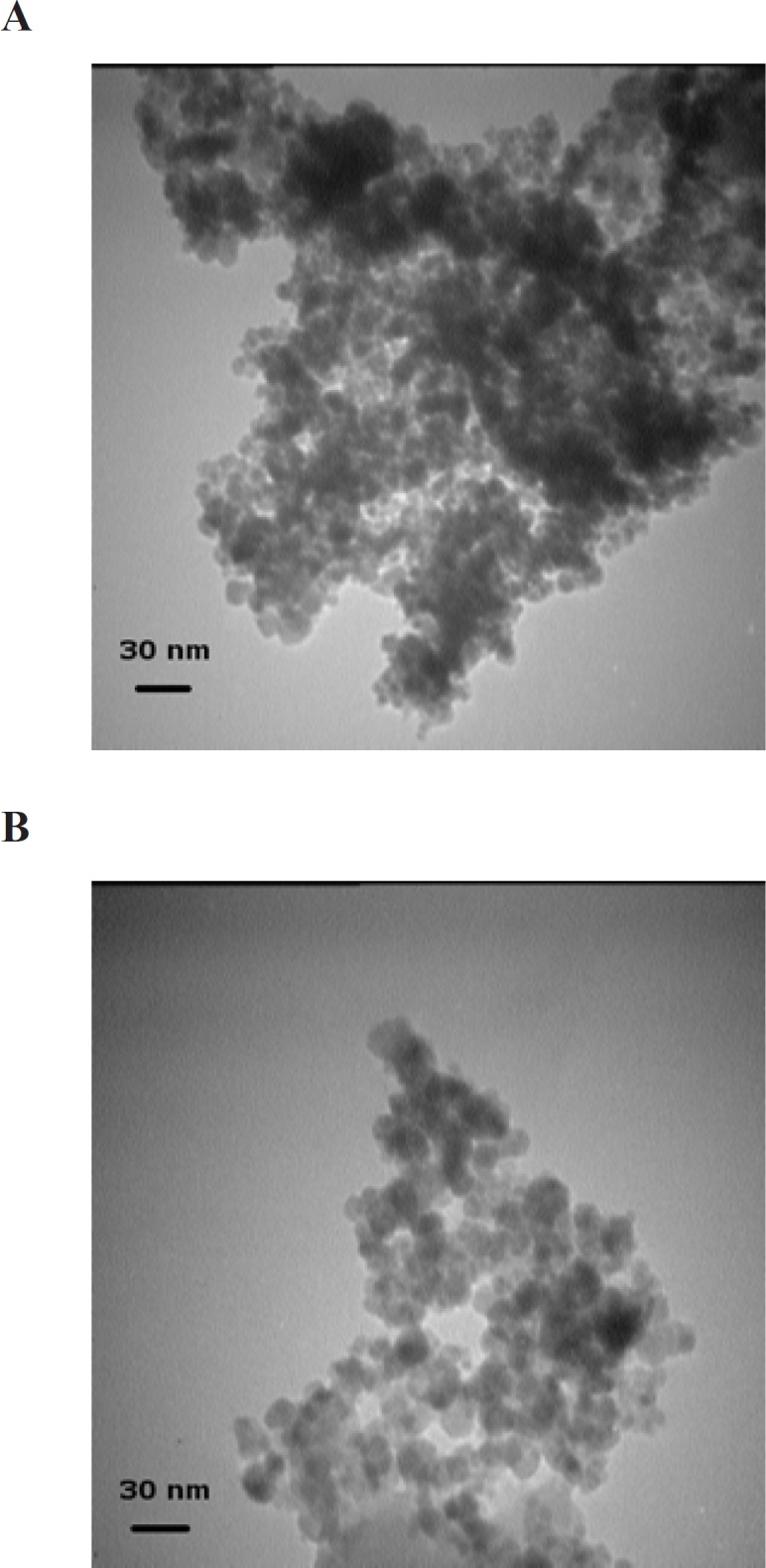
TEM micrographs of Fe_3_O_4_ (A) and Fe_3_O_4_@SiO_2_ (B) NPs. (A) Fe_3_O_4_ NPs were prepared by FeCl_2_ and FeCl_3_ coprecipitation under alkaline conditions. (B) Fe_3_O_4_@SiO_2_ NPs were prepared using the method described in the Experimental section (initial TEOS concentration 0.1 % v/v

Typical TEM images of Fe_3_O_4_ and Fe_3_O_4_@SiO_2_ NPs prepared using our simplified method are shown in Figure 1. The mean diameter of Fe_3_O_4_ NPs is about 8 nm which is in accordance with previous reports (6, 8, 18). Fe_3_O_4_@SiO_2_ NPs, as expected, are larger with an average diameter of ca. 18 nm. The whole synthesis process can be completed in less than 12 h and it does not include any pretreatment or additional steps. This was achieved by introducing a small change in the order and rate at which reagents are added to the mixture so that NaOH is added in the last step over a period of 2 h.

**Table 1.** Yield and quality of genomic DNA samples extracted from different bacterial strains using Fe_3_O_4_@SiO_2_ NPs and a commercial genomic DNA extraction kit. Results are shown as mean ± standard error of the mean

**Bacterial strains**	**Fe**_3_ **O**_4_ **@SiO**_2_ ** NPs**		**EZ-10 Bacterial** **DNA Kit**	
	**Yield (μg)**	**A**_260_ **/A**_280_	**Yield (μg)**	**A**_260_ **/A**_280_
*E. coli*	53.70 ± 3.48	1.93 ± 0.02	60.00 ± 2.64	2.00 ± 0.01
*S. epidermidis*	28.00 ± 4.04	1.73 ± 0.05	34.33 ± 2.96	1.85 ± 0.03
*S. aureus*	12.00 ± 1.15	1.74 ± 0.02	14.67 ± 1.55	1.70 ± 0.03
*B. licheniformis*	17.00 ± 1.15	1.80 ± 0.01	15.33 ± 1.20	1.77 ± 0.01
*Y. enterocolitica*	19.67 ± 1.45	1.83 ± 0.02	23.00 ± 2.08	1.85 ± 0.03
*P. aeruginosa*	26.67 ± 0.88	1.86 ± 0.02	21.33 ± 1.86	1.92 ± 0.02

**Figure 2:**
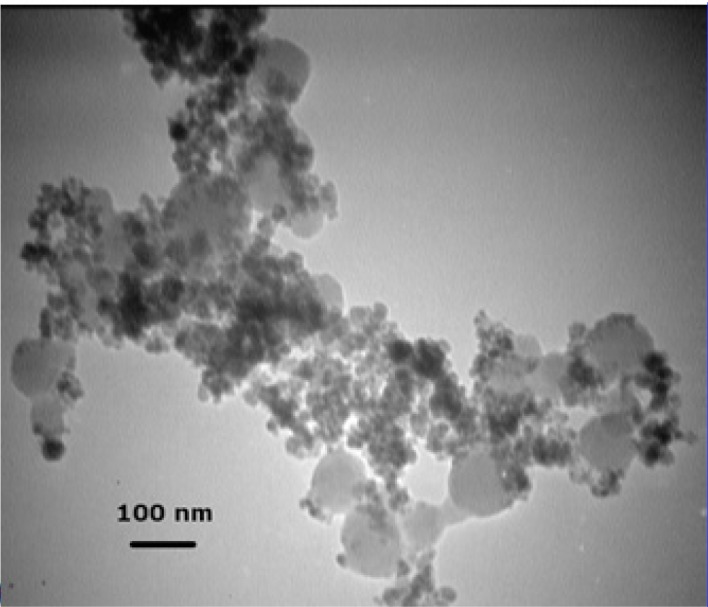
TEM micrograph of Fe_3_O_4_@SiO_2_ NPs prepared using a TEOS concentration of 0.2% (v/v). As described in the text, large homogenous silica particles without a magnetic core are formed at this increased TEOS concentration

**Figure 3.**
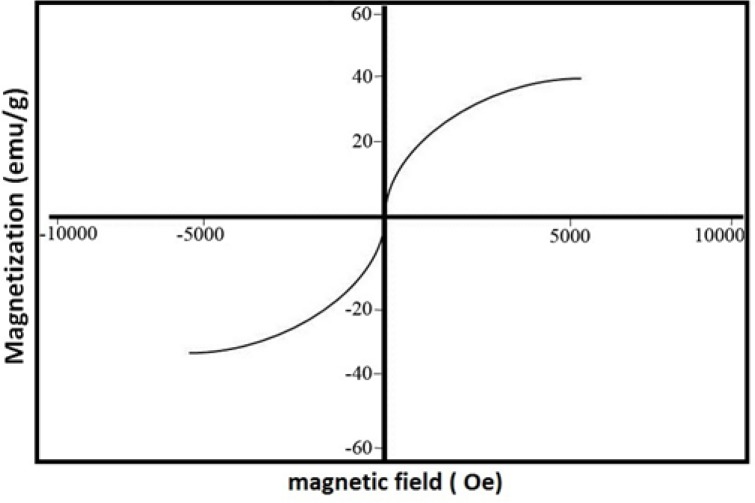
Magnetization curve of Fe_3_O_4_@SiO_2_ NPs recorded at room temperature

**Figure 4:**
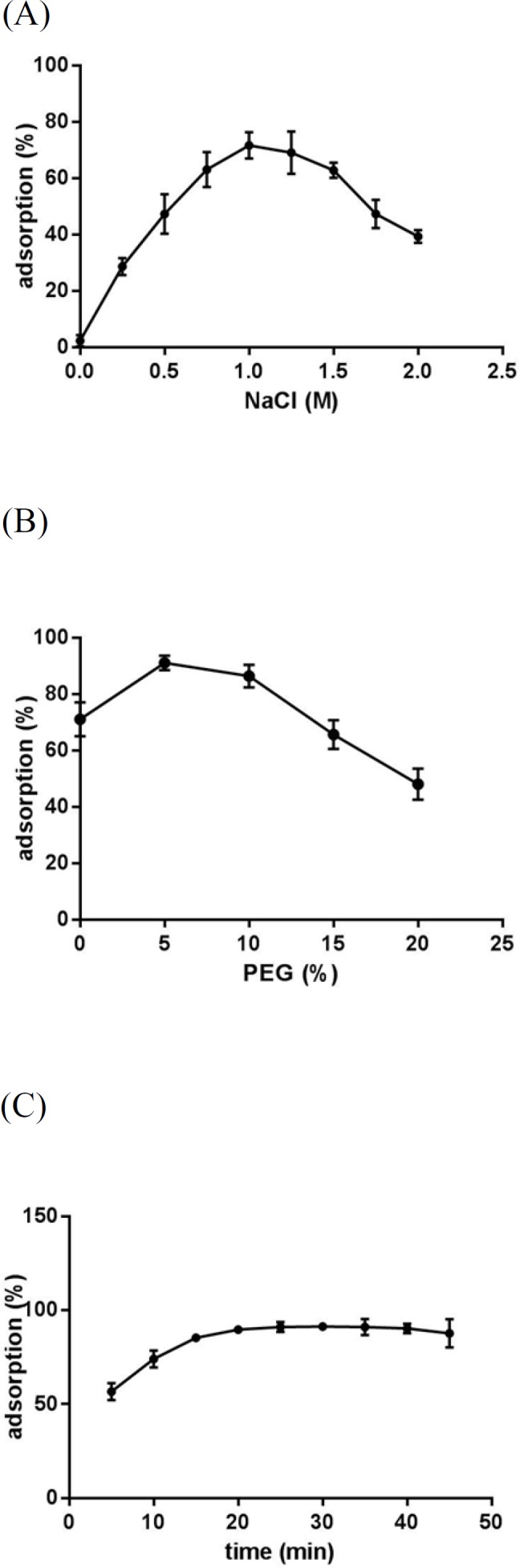
Effect of NaCl concnetration (A), PEG concentration (B) and incubation time (C) on CT-DNA adsorption on Fe_3_O_4_@SiO_2_ NPs. In all experiments, 0.5 mg Fe_3_O_4_@SiO_2_ NPs and 12 μg CT-DNA in a final volume of 100 μL were used. (A) Different concentrations of NaCl were added to 10 mM Tris-HCl buffer (pH 8) and samples were incubated for 30 min at room temperature and 500 rpm. (B) Different concentrations of PEG 8000 were added to 10 mM Tris-HCl buffer (pH 8) containing NaCl (1 M) and samples were incubated for 30 min at room temperature and 500 rpm. (C) Samples prepared in 10 mM Tris-HCl buffer (pH 8) containing NaCl (1 M) and PEG 8000 (5% w/v) were incubated at room temperature and 500 rpm for different time periods

**Figure 5.**
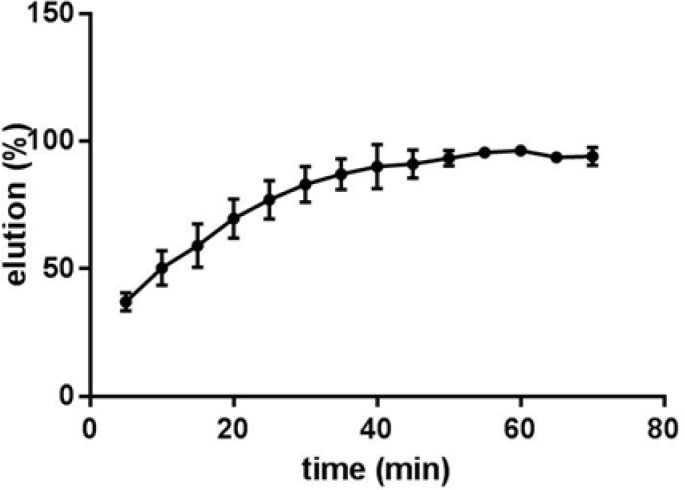
Effect of incubation time on the elution of CT-DNA from Fe_3_O_4_@SiO_2_ NPs. Samples containing 0.5 mg Fe_3_O_4_@SiO_2_ NPs with bound DNA from binding experiments in 100 μL 10 mM TE buffer (pH 8.0) were incubated for different time periods at 50 °C

**Figure 6:**
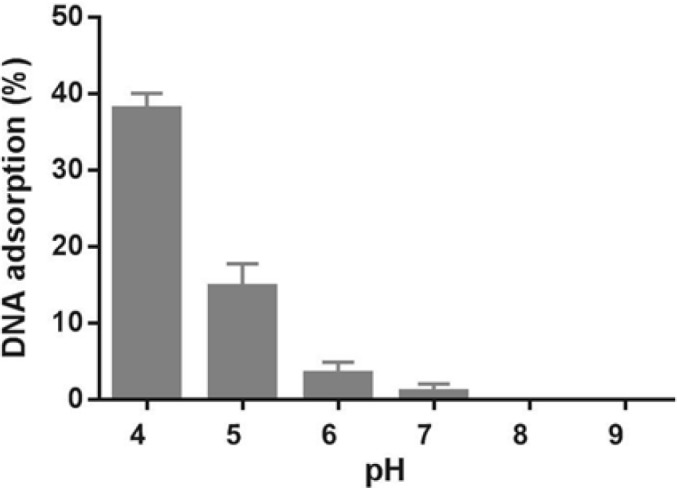
Effect of pH on CT-DNA adsorption on Fe_3_O_4_@SiO_2_ NPs. Samples containing 0.5 mg Fe_3_O_4_@SiO_2_ NPs and 12 μg CT-DNA in 100 μL buffer (10 mM citrate, pH 4-6; 10 mM Tris-HCl, pH 7-9) were incubated at room temperature and 500 rpm for 30 min

**Figure 7:**
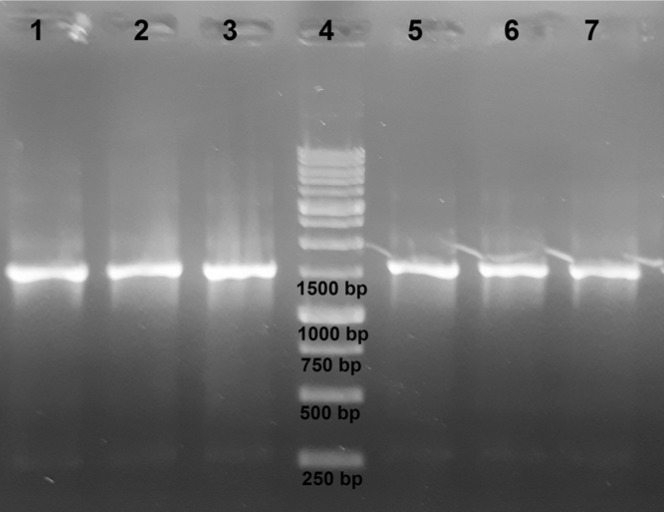
Agarose gel electrophoresis of PCR products. Extracted genomic DNA samples were amplified using 16S rDNA gene-specific primers. From left to right: *E. coli*, *S. epidermidis*, *S. aureus*, DNA ladder, *B. licheniformis*, *Y. enterocolitica*, *P. aeruginosa*

Fe_3_O_4_@SiO_2_ NPs are usually prepared by dispersing Fe_3_O_4_ NPs in a mixture of water, a low molecular weight alcohol (used as cosolvent) and a catalyst (NH_4_OH or NaOH), adding a small amount of TEOS (sometimes in batches) and stirring the mixture for 6 to 48 h at room temperature (4, 6, 8, 9, 14). When untreated Fe_3_O_4_ NPs are used, large aggregates form upon addition of TEOS (6, 8, 9). It has been suggested that the lower pH of the reaction mixture compared to that of the medium in which Fe_3_O_4_ NPs were originally dispersed and the increase in the ionic strength of the mixture resulting from the hydrolysis and condensation of TEOS units are the major contributors to this phenomenon (8). This means that, ironically, the same process that leads to the deposition of a silica coat on Fe_3_O_4_ NPs (and thus increases their stability in aqueous solutions) is also (indirectly) responsible for their flocculation. Whether coating or flocculation occurs is determined by the kinetics of the two pathways. Apparently, flocculation cannot be prevented simply by decreasing the rate of polymerization as this would also affect coating kinetics. One should also note that coating Fe_3_O_4_ NPs using the Stober method is a slow process that requires several hours to complete (6) and thus further decreasing the reaction rate would not be favorable from an economic point of view either. A better approach would be to minimize nucleation while sustaining the rate of growth (6, 9). 

The formation of silica during the Stober method occurs via two distinct but related processes: nucleation and growth (19, 20). Nucleation is the process during which the first insoluble species, probably doubly hydrolyzed TEOS monomers, form and precipitate (21). Growth, on the other hand, results from both the addition of newly hydrolyzed monomers to these nuclei and the aggregation of small particles to form larger ones (not to be confused with Fe_3_O_4_ NP aggregation) (20, 22). Although these two processes are closely related and cannot be completely separated, their relative contribution to the whole reaction can be modified to some extent by manipulating reaction conditions (23). The choice between nucleation and growth is determined by the rates at which hydrolyzed monomers are produced and consumed (20). If these hydrolyzed monomers are produced at a higher rate than they can be incorporated into existing particles, new nuclei are formed. Otherwise, simple growth occurs. Therefore, nucleation can be minimized by either increasing the concentration of seed particles or reducing TEOS hydrolysis rate (20). Increasing the concentration of Fe_3_O_4_ NPs, however, would also promote flocculation. So there is a limit to the concentration of Fe_3_O_4_ NPs beyond which flocculation would occur. Philipse *et al*. showed that in order for silica growth to outpace flocculation the concentration of seed particles should be kept below 12 mg/L (8). However, other teams were able to achieve comparable results at concentrations of as high as 0.4 to 1 g/L (9, 10, 24). Our preliminary experiments at a range of 0.5-5 g/L revealed that a seed concentration of 1 g/L is acceptable. We were thus left with only one option: reducing the rate of TEOS hydrolysis. However, TEOS hydrolysis is the rate-limiting step in silica formation during the Stober process (25). Any factor that negatively affects this step would also hamper the coating process. We hypothesized that by first reducing the rate of hydrolysis to allow the formation of a thin silica layer around Fe_3_O_4_ NPs under suboptimal conditions for a short period and then, after NPs have been stabilized enough to withstand variations in medium ionic strength, gradually increasing the rate to promote further silica deposition it may be possible to address this problem. The rate of TEOS hydrolysis is determined by the concentrations of TEOS and the catalyst (25). Although theoretically either one can be used to control hydrolysis rate, in practice it is much easier to use the catalyst as it is not consumed during the process. The reaction thus would start at low pH and (relatively) high TEOS concentrations. In principle, this stage is analogous to the pretreatment step described by Philipse *et al*. (8). However, since in that case sodium silicate was used instead of TEOS, nucleation was avoided by increasing medium pH. It has been shown that using NaOH instead of NH_4_OH as the catalyst allows for more precise control over the process (14). It is also used at lower concentrations (10-20 mM) (14). So we decided to use NaOH. Several experiments were performed to determine the optimal final concentration of NaOH (10-200 mM) and the time span over which it should be added (5-120 min) to the mixture. We obtained the best results with a 2 M solution added in ten 0.1 mL batches over 2 h. The first three batches (0.3 mL) can be added at once without any detrimental effect. At higher NaOH concentrations or when NaOH was added over a shorter period visible aggregation occurred. We also notice that even when NaOH concentration is gradually increased, the initial concentration of TEOS should be carefully adjusted as even small deviations results in homogeneous nucleation. For example, Figure 2 shows the electron micrograph of Fe_3_O_4_@SiO_2_ NPs prepared using an initial TEOS concentration of 0.2 % (v/v). Silica spheres without a magnetic core can clearly be seen. The large size of the majority of these silica particles indicates that they probably formed during the early stages when TEOS concentration was highest. Reducing TEOS concentration to 0.1 % (v/v) eliminates this problem (Figure 1B).

There appears to be a critical concentration above which homogenous nucleation occurs at considerable rates. In our method this concentration appears to lie somewhere between 0.1 and 0.2% (v/v). Philipse *et al*. noticed a similar effect above 0.16% (v/v) despite the fact that their experiments were performed under drastically different conditions (8). Other research groups reported noticeable homogenous nucleation at a TEOS concentration of 0.5 but not 0.08% (v/v) (26). Nevertheless, we believe that generalization should be avoided as there is a complex relationship between the concentrations of different reagents in the Stober process (4). It has been suggested that limiting the initial concentration of TEOS may reduce the size of Fe_3_O_4_@SiO_2_ NPs (4). In fact, some researchers have employed this effect to control the final size of NPs (4, 18). As stated above, the average diameter of Fe_3_O_4_@SiO_2_ NPs prepared by our method is about 18 nm which is somewhat smaller than those reported in some studies (5, 8, 10, 27). However, Fe_3_O_4_@SiO_2_ NPs with a silica coat of only 2-5 nm have also been prepared and successfully used in biomedical applications (6, 28). Some researchers even argue that small size may actually provide some advantages such as improved *in-vivo* compatibility (6). Increasing the thickness of the silica coat also negatively affects the magnetic properties of the NPs (4, 18). The magnetization curve of Fe_3_O_4_@SiO_2_ NPs prepared by our method is shown in Figure 3. The saturation magnetization of the particles is 37.8 emu/g which is relatively higher than those reported in the literature (4, 18). However, if NPs of larger size are required, a sequential seeded approach can be used to obtain appropriate results (20). We did not explore this possibility since, as described below, Fe_3_O_4_@SiO_2_ NPs produced using this method demonstrated satisfactory properties.

In order to investigate whether Fe_3_O_4_@SiO_2_ NPs prepared using the method describe in this paper possess the characteristics required for biomedical applications we evaluated their performance for isolating genomic DNA from bacterial samples. We began by determining the optimum binding and elution conditions for DNA using a standard CT-DNA solution. Results are shown in Figure 4. Maximum adsorption was achieved in 10 mM Tris-HCl buffer (pH 8.0) containing NaCl (1 M) and PEG 8000 (5% w/v). It was also noticed that incubation periods longer that 20-35 min did not result in higher yields. Under optimal conditions, ca. 90% of CT-DNA was adsorbed onto the surface of Fe_3_O_4_@SiO_2_ NPs. For elution experiments the only factor that required optimization was the incubation time as the composition of the elution solution is quite simple and usually consists of TE buffer or water (27, 29-31). As shown in Figure 5, elution in 10 mM TE buffer (pH 8.0) was complete after 50-60 min. It should be noted that although in theory adsorption and elution can be achieved simply by adjusting the pH of the medium (32), which is also consistent with our results presented in Figure 6, we noticed that especially at lower bacterial loads when larger amounts of starting materials were required this method failed to produce satisfactory results (data not shown). We then used the optimized protocol to extract genomic DNA from 5 different bacterial strains and compared the result with those obtained using a commercial kit (Table 1). A statistical analysis of the results using two-sample *t*-test showed no significant (*p*-value < 0.05) differences between the two procedures for any of the strains.

Genomic DNA samples are frequently used to isolate a specific gene. It is thus important that the extracted DNA samples do not contain any impurities that may inhibit PCR; otherwise, time-consuming purification steps may be required. To investigate whether DNA samples prepared using Fe_3_O_4_@SiO_2_ NPs can be directly used for PCR amplification without further processing, we used extracted DNA samples to amplify a region of the 16S rDNA gene. As shown in Figure 7, all samples were successfully amplified.

The results presented in this paper suggest that the requirement for Fe_3_O_4_ pretreatment in the preparation of Fe_3_O_4_@SiO_2_ NPs using the Stober process can be bypassed by introducing minor modifications to the reaction conditions so that a short period of slow silica growth is allowed to proceed in the absence of noticeable nucleation. This goal can be achieved by reducing the rate of TEOS hydrolysis by limiting the initial concentration of NaOH in the reaction mixture and then gradually increasing its concentration as the reaction proceeds. Fe_3_O_4_@SiO_2_ NPs produced by this method show good physicochemical properties can be used for biological applications such as genomic DNA extraction with a separation quality comparable to that of commercial kits.
